# Molecular Modeling Approaches Can Reveal the Molecular Interactions Established between a Biofilm and the Bioactive Compounds of the Essential Oil of *Piper divaricatum*

**DOI:** 10.3390/molecules27134199

**Published:** 2022-06-29

**Authors:** Jorddy Neves Cruz, Mozaniel Santana de Oliveira, Eloisa Helena de Aguiar Andrade, Rafael Rodrigues Lima

**Affiliations:** 1 Laboratory of Functional and Structural Biology, Institute of Biological Sciences, Federal University of Pará, Belem 66075-110, PA, Brazil; rafalima@ufpa.br; 2 Adolpho Ducke Laboratory, Museu Paraense Emílio Goeldi, Belem 66040-170, PA, Brazil; mozaniel.oliveira@yahoo.com.br (M.S.d.O.); eloisa@museu-goeldi.br (E.H.d.A.A.)

**Keywords:** gelatin, biofilm, molecular interactions, in silico, molecular modeling

## Abstract

Molecular modeling approaches are used in a versatile way to investigate the properties of diverse organic and inorganic structures such as proteins, biomolecules, nanomaterials, functionalized nanoparticles, and membranes. However, more detailed studies are needed to understand the molecular nature of interactions established in gelatin biofilms impregnated with bioactive compounds. Because of this, we used computational methods to evaluate how the major compounds of *Piper divaricatum* essential oil can interact with the gelatin biofilm structure. For this, we used as inspiration the paper published, where various properties of the essential oil impregnated gelatin biofilm *P. divaricatum* are reported. After our computer simulations, we related our molecular observations to biofilm’s structural and mechanical properties. Our results suggest that the major compounds of the essential oil were able to interrupt intermolecular interactions between the chains of the biofilm matrix. However, the compounds also established interactions with the amino acid residues of these chains. Our molecular analyses also explain changes in the structural and mechanical properties of the essential oil-impregnated biofilm. These results can support the planning of functional packaging impregnated with bioactive compounds that can protect food against microorganisms harmful to human health.

## 1. Introduction

The use of food packaging based on synthetic polymers derived from petroleum has contributed to a significant increase in environmental pollution over the decades. This risks an entire marine and terrestrial production chain and generates an ecological imbalance [1,2,3].

Foods that use plastic packaging can be contaminated by microplastics and pose a health risk [4]. These particles may be potential immunostimulants at the cellular level, inducing the production of cytokines and chemokines [5].

With the advancement of food science and technology, new methods are developed to preserve food characteristics, such as biodegradable packaging, which can be a viable alternative to replace packaging produced from petroleum-derived polymers [6,7].

Another packaging alternative that can be considered ecologically correct is those produced from biodegradable biofilms derived from fish or renewable polymers. Packaging of this nature can positively impact the environment as they suffer from environmental degradation and are made from reusable waste [8].

The proteins that make up biodegradable biofilms that can be applied to packaging production are low-cost and readily available biopolymers obtained from 45 accessible sources of porcine, bovine, and fish gelatin [9]. The main characteristics of these gelatin biofilms include their mechanical strength, thermal stability, optical opacity, and surface roughness [10,11].

In addition, gelatines used as packaging can be important vehicles for dispersing bioactive compounds to preserve food quality. These compounds impregnated in biofilms, among other properties, may have antioxidant and antimicrobial action. These properties make packaging functional, increase food shelf life, and provide greater protection against microorganisms that can degrade food [12,13].

As an example, we have the gelatines incorporated with essential oils of *Bergamot orange* and *Cymbopogon flexuosus* that were able to inhibit the growth of *Escherichia coli*, *Listeria monocytogenes*, *Staphylococcus aureus,* and *Salmonella typhimurium* [14]. Additionally, the essential oils of *Origanum vulgare* and *Lavandula officinalis* impregnated in packaging were effective in inhibiting *E. coli* and *S. aureus*, demonstrating that the incorporation of natural products, especially essential oils, can be an alternative to improve the functional properties of biofilms and coatings of commercial products [15,16,17] due to their diverse biological properties [18,19,20,21]. 

Studies on incorporating volatile molecules to improve the functional characteristics of gelatins are essential [22]. However, it is necessary to disseminate scientific knowledge about their interaction mechanisms. Thus far, this has been done very superficially. A possible interaction mechanism has been proposed, but the interactions are displayed and discussed in a very simplified and inconclusive way [23,24].

In this paper, we utilize molecular modeling approaches to fill the gap related to the molecular investigation of the structure of gelatin biofilms impregnated with bioactive compounds. This methodology has been used to investigate molecular systems of different types [25,26,27,28,29]. As a basis for our studies, we used the structure proposed by Hanani et al. (2014) [30] to build the gelatin, and the work of Albuquerque et al. (2020) [31] inspired us to carry out our molecular investigations. Albuquerque et al. (2020) impregnated [16] a gelatine biofilm with supercritical fluid with the essential oil of *Piper divaricatum* rich in methyl eugenol (57.02%), eugenyl acetate (14.75%), eugenol (13.27%) and β-elemene (6.39%). Here, we evaluate and compare our results obtained in silico with those observed experimentally by the authors. We consider, among other things, how the bioactive compounds influence the physicochemical, structural, and biological properties of the oil-impregnated gelatin biofilm. Thus, we report for the first time in the literature in a detailed and in-depth manner, using computational methods, the impact of impregnating gelatin-based biofilms with volatile molecules.

## 2. Results and Discussions

### Molecular Analysis of the Biofilm Matrix

**Figure 1:**
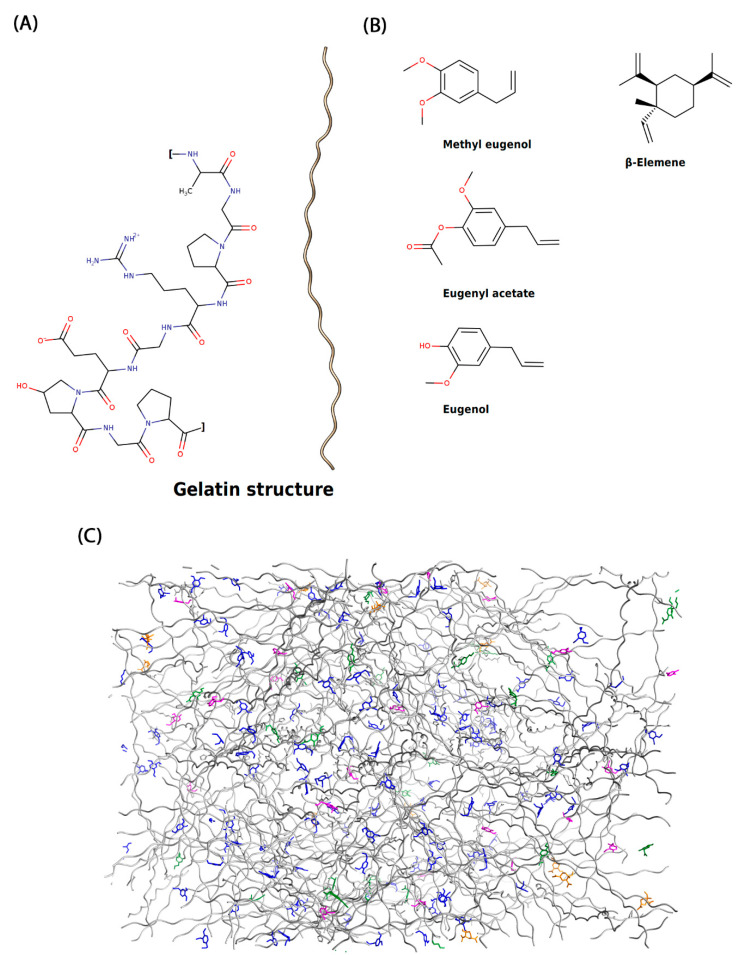
(**A**) Molecular structure of gelatin where the typical amino acids of the structure and its three-dimensional structure with the elemental composition of 50.5% carbon, 25.2% oxygen, 17% nitrogen, and 6.8% hydrogen are displayed. (**B**) Major EO compounds have been impregnated into gelatin. The methyl eugenol, eugenyl acetate, eugenol, and β-elemene represent 48.01, 22.55, 10.52, and 7.38% of the total composition of the EO obtained using extraction with supercritical CO_2_ fluid at a temperature of 100 °C and a pressure of 100 bar. (**C**) Structural complex showing the arrangement of EO components in the gelatin matrix.

The molecular structure of gelatin impregnated with the major compounds of EOs can be seen in Figure 1.

**Figure 2:**
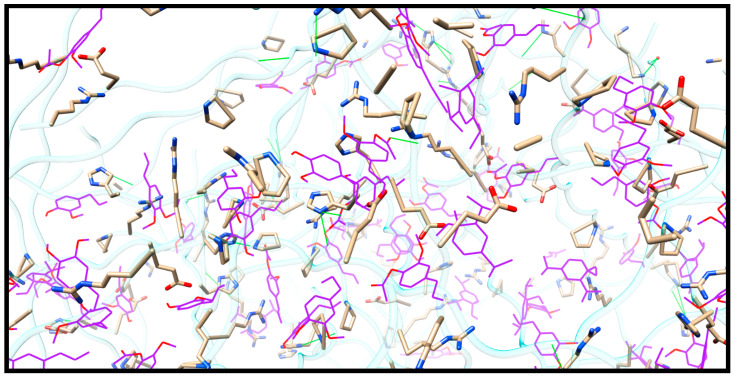
Enlarged view of the EO-impregnated biofilm, where the side chains of the amino acids that interact with the EO compounds are represented (color purple). The amino acid side chains are represented by sticks, while the EO compounds are represented on the wire. The green lines represent hydrogen bonds. Color coding to represent atoms: oxygen is colored red, and nitrogen, blue.

The major EO compounds were dispersed in the gelatin matrix. These compounds interacted non-covalently with the polypeptide chain that forms the biofilm. Chemical interactions were essentially hydrophobic in nature, as can be seen in Figure 2.

**Figure 3:**
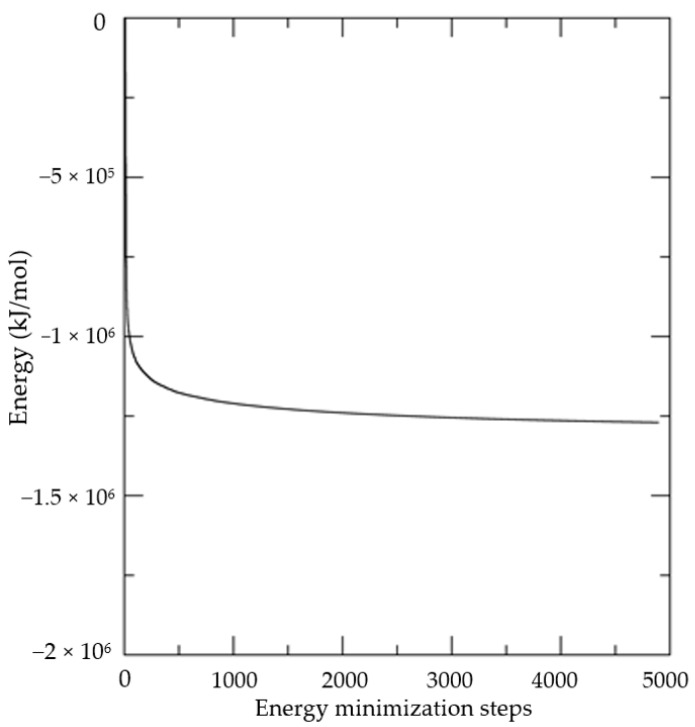
Complex minimization energy.

Complex energy analyses were performed to obtain quantitative parameters and assess whether something strange occurred along with the molecular dynamics simulation (MD simulations). Figure 3 demonstrates the nice, steady convergence of the complex energy throughout the MD simulation. This indicates that the protocol used was able to smooth the components of the complex to achieve a thermodynamically favorable molecular structure.

**Figure 4:**
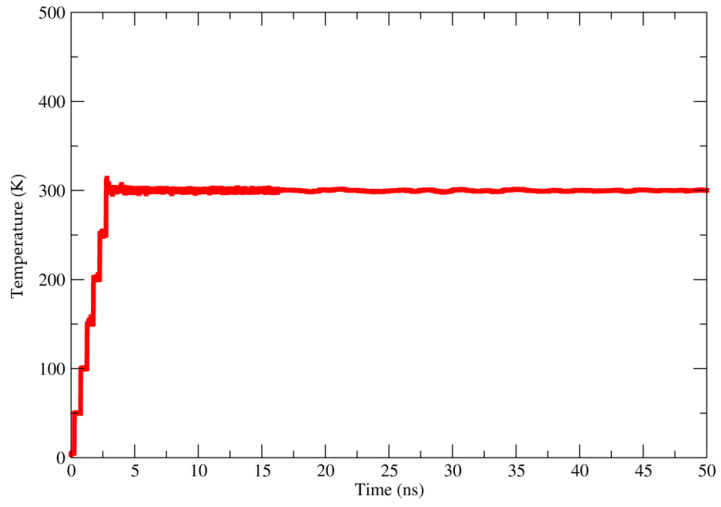
Complex temperature over the MD simulation.

The temperature of the complex was monitored to assess whether the heating was performed gradually and smoothly. As shown in Figure 4, the temperature of the complex after 2.5 ns stabilized at approximately 300 K. This sounds good, as 300 K is a temperature found in typical living environments, simulating the exposure sites of biofilms impregnated with bioactive compounds.

**Figure 5:**
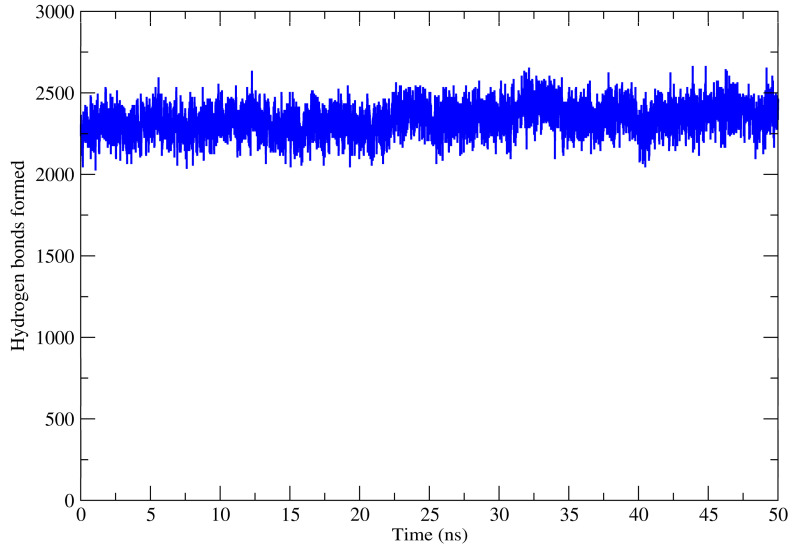
Hydrogen bonds formed throughout the MD simulations.

In the EO-free gelatin matrix, the side chains of amino acids interact with the side chains of neighboring amino acids. These non-covalent interactions can be hydrogen bonds, dipole-dipole, or ionic bonding (salt bridges). Throughout the MD simulations, the hydrogen bonds were monitored (Figure 5). The registration of hydrogen bonds was performed for all interactions established, that is, all interactions formed between biofilm compounds. 

These interactions are closely related to the structure of the gelatin matrix. Thus, if these interactions are interrupted or compounds are added to this polypeptide network, their incorporation may favor or impair the maintenance of the biofilm’s polypeptide matrix. Thus, the molecular interactions/gelatin matrix can influence the biofilm’s structural, physicochemical, and biological properties.

SEM micrographs showed that the matrix of the control biofilm (biofilm without EO) was altered after EO impregnation [31]. In our molecular modeling approaches, we observed that the EO molecules were accommodated in the interchain spaces of the gelatin matrix. Furthermore, these molecules were able to interrupt some intermolecular interactions between the polypeptide chains. The lost interactions were replaced by others established with the EO molecules. Thus, changes in the properties of the oil biofilm were observed.

Oil impregnation promoted changes in the biofilm’s structural, mechanical, and antioxidant properties. The essential oil compounds increased the thickness of the oil biofilm in relation to the control biofilm by approximately 0.015 mm. In addition, EO altered the physical properties of the biofilm, making it less resistant to traction and increasing its elongation capacity. 

This is justified from the molecular view of the complex because the EO molecules occupy an essentially larger space within the biofilm’s polypeptide chains. This explains the biofilm’s increase in thickness and greater capacity for elongation. The non-rupture of this biofilm during its extension may be related to the intermolecular interactions that the oil compounds can establish between the chains. It would be as if these compounds participated in the composition of the biofilm as links between the chains, allowing them to be elongated to a specific size, with the structure breaking from the breakage of the polymeric chains and loss of the bonds and molecular interactions between the atoms of the chain itself and those established with the oil compounds.

Another observation we can provide from our computational studies is that the EO compounds disrupted interchain hydrogen interactions within the biofilm structure, weakening the intermolecular forces that maintain the matrix cohesion. This directly impacted the ability of the gelatin matrix to remain resistant to traction, as the interactions that kept one chain more tightly bound to another became scarcer. However, this increased the plasticity of gelatin, increasing its elongation capacity.

According to Albuquerque et al. (2020) [31], the control biofilm has a more cohesive and denser matrix than the oil biofilm structure. Thus, the control biofilm matrix is more robust and less thick. The impregnation of EO induces a greater distance between its chains in the gelatin to allow the entry and permanence of the EO molecules in its structure. In this way, the gelatin matrix becomes less cohesive, allowing an increase in the mobility of the chains. These changes influenced the thickness of the oil-biofilm.

## 3. Materials and Methods

### Molecular Modeling Approaches

The molecular structure of gelatin was designed according to its typical amino acid composition (-Ala-Gly-Pro-Arg-Gly-Glu-4Hyp-Gly-Pro-) proposed by Nur Hanani et al. (2011) [30]. The primary amino acid sequence was drawn in GaussView 5 software [32] and submitted to an energy minimization protocol in the sander module of the AMBER 16 [33,34,35]. The energy of the typical gelatin amino acid structure was minimized to obtain energetically favorable molecular structures in conformations close to those observed experimentally. For this, 5000 cycles were performed using the steepest descent method and conjugate gradient algorithm. The major compounds (methyl eugenol, eugenyl acetate, eugenol and β-elemene) of the essential oil of *P. divaricatum* were designed using the software GaussView 5 [32], and its molecular structure was optimized using B3LYP/6-31G [36,37,38] with Gaussian 09 software [38,39]. The biofilm-EO model was built using PACKMOL software [40,41]. These compounds were added to the gelatin model taking into account their percentages found in the EO composition. That is, in the total composition of the constructed molecular system, it is possible to find 48.01% de methyl eugenol, 22.55% de eugenyl acetate, 10.52% eugenol, and 7.38% de β-elemene. MD simulations of 50 ns were performed at constant volume by heating the systems up to 300 K. The Particle Mesh Ewald method [42] was used for the calculation of the electrostatic interactions, and the bonds involving hydrogen atoms were restricted with the SHAKE algorithm [43]. The temperature control was performed with a Langevin thermostat [44] within a collision frequency of 2 ps^−1^.

## 4. Conclusions

The EO compounds altered the network of intermolecular interactions of the polypeptide chains that make up the gelatin matrix. The compounds interrupted some interchain interactions but were also able to establish new interactions with the biofilm chains. Changes in molecular interactions and their influence on the structure of the gelatin matrix promoted changes in the physicochemical, structural, and biological properties of the biofilm impregnated with EO. In addition, the present paper can serve as a basis for future strategies for selecting molecules with potential physical activity that can be incorporated into biofilms to be used as functional packaging capable of preserving food quality.

## Data Availability

Not applicable.

## Sample Availability

Simulation files are available from the authors.
